# Combined Burden of Heart Failure and Arterial Hypertension as Predictors of Adverse Outcomes in Hospitalized COVID-19 Patients

**DOI:** 10.3390/jcm15031143

**Published:** 2026-02-02

**Authors:** Ana-Maria Pah, Ana-Olivia Toma, Camelia-Oana Muresan, Diana-Maria Mateescu, Ioana-Georgiana Cotet, Luchian Iancu-Ciorbagiu, Adrian-Cosmin Ilie, Daian Ionel Popa, Dragos-Mihai Gavrilescu, Stela Iurciuc, Maria-Laura Craciun, Simina Crisan, Adina Avram

**Affiliations:** 1 Cardiology Department, “Victor Babes” University of Medicine and Pharmacy, Eftimie Murgu Square 2, 300041 Timisoara, Romania; anamaria.pah@umft.ro (A.-M.P.);; 2 Discipline of Dermatology, “Victor Babes” University of Medicine and Pharmacy, 300041 Timisoara, Romania; toma.olivia@umft.ro; 3 Center for the Morphologic Study of the Skin (MORPHODERM), Victor Babeș University of Medicine and Pharmacy Timișoara, 300041 Timișoara, Romania; 4 Legal Medicine, Timisoara Institute of Legal Medicine, 300041 Timisoara, Romania; 5 Ethics and Human Identification Research Center, “Victor Babes” University of Medicine and Pharmacy, Eftimie Murgu Square 2, 300041 Timisoara, Romania; 6 Discipline of Forensic Medicine, Bioethics, Deontology, and Medical Law, Department of Neuroscience, “Victor Babes” University of Medicine and Pharmacy, 300041 Timisoara, Romania; 7 Doctoral School, Department of General Medicine, “Victor Babes” University of Medicine and Pharmacy, Eftimie Murgu Square 2, 300041 Timisoara, Romania; 8 Railway Clinical Hospital (Spitalul Clinic CFR), Strada Gării 1, 300166 Timisoara, Romania; 9 Department of Public Health and Sanitary Management, “Victor Babes” University of Medicine and Pharmacy, Eftimie Murgu Square 2, 300041 Timisoara, Romania; 10 Centre for Translational Research and Systems Medicine, Faculty of Medicine, “Victor Babeș” University of Medicine and Pharmacy, Eftimie Murgu Square No. 2, 300041 Timișoara, Romania; 11 Research Center for Medical Communication, “Victor Babes” University of Medicine and Pharmacy, Eftimie Murgu Square 2, 300041 Timisoara, Romania; daian-ionel.popa@umft.ro; 12 Department of Orthodontics, Dental District, Strada Zăgazului Nr. 3, One Floreasca Vista, Sector 1, 014261 Bucharest, Romania; 13 Department of Internal Medicine I, “Victor Babes” University of Medicine and Pharmacy, Eftimie Murgu Square 2, 300041 Timisoara, Romania

**Keywords:** COVID-19, arterial hypertension, heart failure, cardiovascular multimorbidity, in-hospital mortality, intensive care admission, interleukin-6

## Abstract

**Background**: Cardiovascular comorbidities are major determinants of poor outcomes among patients admitted with COVID-19. However, the prognostic role of arterial hypertension alone remains uncertain. Little is known about the cumulative impact of concomitant hypertension and heart failure. This study assessed whether the combined burden of arterial hypertension and pre-existing heart failure identifies a high-risk phenotype for adverse in-hospital outcomes among COVID-19 patients. **Methods**: In this retrospective, real-world cohort study, 395 consecutive adults hospitalized with confirmed COVID-19 at a single infectious diseases center between March 2020 and December 2024 were included. We categorized patients into three cardiovascular phenotype groups: no hypertension or heart failure (*n* = 23), hypertension without heart failure (*n* = 193), and concomitant hypertension and heart failure (*n* = 178). The primary outcome was in-hospital all-cause mortality, while ICU admission served as a secondary outcome, invasive mechanical ventilation, and length of hospital stay. Multivariable logistic regression included age, sex, BMI, diabetes mellitus, and vaccination status to evaluate independent associations between the cardiovascular risk group and outcomes. **Results**: Overall in-hospital mortality was 7.3% (29/395). Mortality increased stepwise across the cardiovascular risk groups: 8.7% in patients without hypertension or heart failure, 3.1% in those with hypertension only, and 11.8% in patients with concomitant hypertension and heart failure (*p* = 0.004). In adjusted analyses, concomitant hypertension and heart failure were linked to higher adjusted odds of in-hospital death than no cardiovascular disease (odds ratio, 3.49; 95% confidence interval, 1.46–8.35). Isolated hypertension was not significantly associated with mortality. ICU admission and length of hospital stay also increased with cumulative cardiovascular burden. Patients with combined hypertension and heart failure showed more pronounced inflammatory and renal abnormalities at admission. **Conclusions**: Among hospitalized COVID-19 patients, the coexistence of arterial hypertension and heart failure identifies a vulnerable cardiovascular phenotype associated with higher in-hospital mortality and resource use than either no cardiovascular disease or hypertension alone. These findings support evaluating cardiovascular comorbidities cumulatively rather than in isolation. These findings are exploratory and require external validation in independent, larger multicentre cohorts. Findings may support careful use for short-term risk stratification and closer monitoring strategies during COVID-19 hospitalization.

## 1. Introduction

Coronavirus disease 2019 (COVID-19) encompasses the spectrum of clinical illness associated with infection by severe acute respiratory syndrome coronavirus 2 (SARS-CoV-2) [1,2]. Despite progress in prevention and adjunctive management, COVID-19 has generated a sustained global health burden. It continues to cause severe disease and fatal in-hospital outcomes among hospitalized patients [2]. While pulmonary involvement dominates the acute presentation, strong evidence shows that pre-existing cardiovascular vulnerability shapes the clinical course and outcomes [3,4].

Cardiovascular disease is a major prognostic modifier in COVID-19. ICU admission is more frequent, and mortality is higher in patients with cardiovascular comorbidities [5,6]. SARS-CoV-2 does not only affect the lungs. It also triggers systemic inflammation and endothelial injury. This combination promotes thrombosis and can destabilize pre-existing cardiovascular disease [7,8].

In clinical practice, hypertension and heart failure are often seen together, particularly in elderly patients [9,10]. These conditions share key biological features. These include chronic low-grade inflammation, endothelial dysfunction, arterial stiffness, and neurohormonal activation [11,12]. These pathways overlap with mechanisms implicated in severe COVID-19 [12]. Therefore, patients with established cardiovascular disease may have reduced physiological reserve during acute SARS-CoV-2 infection.

Although arterial hypertension is frequently reported among hospitalized COVID-19 patients, its independent prognostic role remains debated. This is especially true after adjustment for age and multimorbidity [13]. In contrast, meta-analytic evidence supports an association between hypertension and increased COVID-19 severity and mortality. These findings also highlight marked heterogeneity across cohorts [14].

Pre-existing heart failure has been consistently associated with adverse outcomes in COVID-19 [15,16]. Patients with heart failure may deteriorate rapidly. They have limited cardiac reserve, vulnerability to volume overload, arrhythmias, and exaggerated inflammatory and neurohormonal responses [16]. In addition, acute SARS-CoV-2 infection may further aggravate cardiac function. This occurs through hypoxemia, cytokine-mediated injury, endothelial damage, and microvascular thrombosis [17,18].

This combined cardiovascular burden is clinically relevant because arterial hypertension and heart failure commonly co-occur and may reflect a continuum of progressive cardiovascular injury rather than independent entities [9,10]. Evaluating them in isolation may underestimate risk and fail to recognize a subgroup with increased vulnerability who could benefit from intensified monitoring and more tailored management strategies. Furthermore, evidence from Eastern European populations—where cardiovascular disease prevalence is high and healthcare system characteristics differ from Western cohorts—remains comparatively scarce [9].

Despite extensive evidence evaluating arterial hypertension and heart failure separately, the prognostic implications of their combined presence remain insufficiently characterized, particularly in real-world settings with high cardiovascular disease burden. This gap is especially relevant in Eastern Europe, where cardiovascular multimorbidity is highly prevalent and healthcare system constraints may modify outcomes. In addition, it remains uncertain whether cumulative cardiovascular burden retains prognostic relevance in the later pandemic phases, characterized by widespread vaccination and circulation of Omicron and its sublineages.

This study evaluated whether the combined presence of arterial hypertension and pre-existing heart failure is linked to worse short-term in-hospital outcomes for COVID-19 patients. By stratifying patients by cardiovascular burden and analyzing in-hospital mortality, ICU admission, and hospital course, this cohort study aimed to refine risk assessment in an Eastern European setting. To our knowledge, real-world evidence from Eastern European inpatient cohorts quantifying the prognostic impact of this combined phenotype remains limited.

Finally, the pandemic landscape evolved substantially during 2020–2024, with successive SARS-CoV-2 variants (Alpha, Delta, and Omicron sublineages) differing in transmissibility, virulence, and clinical phenotype. Our cohort spans March 2020 to December 2024 and therefore captures multiple pandemic phases; however, comprehensive variant characterization was not routinely available in our setting. Nevertheless, cardiovascular vulnerability driven by cumulative cardiovascular disease may represent a variant-agnostic risk amplifier, as the interaction between chronic cardiovascular dysfunction and systemic inflammatory stress is not lineage-specific. In this context, clarifying whether cumulative cardiovascular burden remains a key determinant of short-term in-hospital outcomes in the vaccinated era is clinically relevant for contemporary and future SARS-CoV-2 surges.

## 2. Materials and Methods

### 2.1. Study Design and Population

The study was conducted at the Department of Infectious Diseases I, Timișoara, Romania. Consecutive adult patients hospitalized with confirmed coronavirus disease 2019 (COVID-19) between March 2020 and December 2024 were screened for eligibility. COVID-19 diagnosis was confirmed by a positive reverse transcription polymerase chain reaction (RT-PCR) or by a validated antigen test, in accordance with national and institutional diagnostic protocols.

Inclusion criteria: (1) Age ≥ 18 years; (2) Hospitalization for acute COVID-19-related illness at the study center; (3) Laboratory-confirmed SARS-CoV-2 infection by RT-PCR or validated antigen test.

Exclusion criteria: (1) Incomplete clinical records regarding cardiovascular comorbidities or the primary outcome (in-hospital mortality); (2) Transfer from another hospital after prolonged admission (>48 h prior to arrival); (3) Admission primarily for reasons unrelated to acute SARS-CoV-2 infection (e.g., elective procedures, trauma).

### 2.2. Data Collection

Data were extracted retrospectively from electronic records using a standardized form. Variables included age, sex, BMI, smoking status, vaccination status, major comorbidities (diabetes mellitus, chronic kidney disease, chronic obstructive pulmonary disease, atrial fibrillation, ischemic heart disease, and others), with emphasis on cardiovascular disease. Chronic cardiovascular medications (e.g., ACE inhibitors or ARBs, beta-blockers, and antihypertensive treatment burden) were collected when available. Laboratory parameters and clinical course during hospitalization were recorded, including ICU admission, mechanical ventilation, hospital length of stay, and in-hospital mortality. Laboratory tests were performed in the hospital’s central laboratory. CBC (Complete blood count) parameters were generated automatically by the hospital laboratory (Sysmex XN-1000, Sysmex Corporation, Kobe, Japan). C-reactive protein (CRP) was measured by immunoturbidimetry as part of routine admission biochemistry testing on the Cobas Integra 400 Plus (Roche Diagnostics, Mannheim, Germany). Interleukin-6 (IL-6) was quantified by ECLIA with the Elecsys^®^ IL-6 kit on a Cobas e601 analyzer (Roche Diagnostics, Mannheim, Germany). D-dimer was measured by immunoturbidimetric assay (STA^®^-Liatest D-Di, Diagnostica Stago, Asnières-sur-Seine, France) on the STA Compact Max analyzer (Diagnostica Stago, Asnières-sur-Seine, France). Serum creatinine was assessed using the kinetic Jaffé method on the Cobas Integra 400 Plus (Roche Diagnostics, Mannheim, Germany). SARS-CoV-2 variant identification and wave classification were unavailable in the database and thus not included.

All data were anonymized prior to analysis, and no patient-identifiable information was accessible to the investigators during data processing.

### 2.3. Definitions of Cardiovascular Conditions

Arterial hypertension was defined as any of the following: a documented diagnosis of hypertension prior to hospital admission in the patient’s medical records, ongoing treatment with antihypertensive medication, or a previous physician diagnosis of hypertension. These criteria were aligned with contemporary European Society of Cardiology (ESC) guidelines to ensure consistency in classification.

Heart failure was defined as a documented clinical diagnosis of heart failure established before hospital admission, regardless of left ventricular ejection fraction phenotype. Where available, the classification included information from echocardiographic reports and New York Heart Association (NYHA) functional class to further characterize the heart failure phenotype. If extensive echocardiographic and NYHA data were unavailable, the diagnosis relied on clinical records and treatment history, specifically including patients with prior heart failure hospitalization or those under chronic treatment for heart failure.

### 2.4. Cardiovascular Risk Stratification

To assess the cumulative cardiovascular burden, patients were stratified into three mutually exclusive groups based on the presence or absence of arterial hypertension and heart failure: (1) No hypertension and no heart failure; (2) Hypertension without heart failure; (3) Concomitant hypertension and heart failure.

This stratification constituted the primary exposure variable for all subsequent analyses.

### 2.5. Short-Term In-Hospital Outcomes

All predefined outcomes were short-term events restricted to the index hospitalization. Post-discharge or post-acute COVID-19 sequelae (long COVID) were not included. The primary outcome was in-hospital all-cause mortality. Secondary outcomes were: (1) admission to the intensive care unit (ICU) during the index hospitalization; (2) requirement for invasive mechanical ventilation; (3) total length of hospital stay (days). Outcomes were assessed from electronic records at hospital discharge or death.

### 2.6. Statistical Analysis

We reported variables as mean (standard deviation) or median with interquartile range (IQR), as appropriate for data distribution. Comparisons across the three cardiovascular risk groups used one-way ANOVA for quantitative variables, and proportions were evaluated with χ^2^ testing or Fisher’s exact test for sparse tables. Admission inflammation was characterized using C-reactive protein (CRP), interleukin-6 (IL-6), and D-dimer, which demonstrated markedly right-skewed distributions and were therefore summarized using median and IQR.

To evaluate the independent association between cardiovascular burden and adverse outcomes, multivariable logistic regression models were constructed. The primary exposure variable was the cardiovascular risk group, with the group without hypertension or heart failure as the reference category. Models were adjusted a priori for clinically relevant confounders, including age, sex, BMI, diabetes mellitus, and vaccination status. Covariates were defined in advance using a clinically driven approach; no automated variable selection procedures (e.g., stepwise selection) were used. We examined multicollinearity by calculating variance inflation factors (VIFs) for the adjusted models, which indicated no problematic collinearity. Because of the limited number of deaths, the model was intentionally parsimonious to reduce overfitting; estimates should therefore be interpreted with caution. Odds ratios (ORs) with 95% confidence intervals (CIs) are provided. Statistical significance was set at *p* < 0.05. Analyses were conducted in SPSS (IBM) for Windows, version 26.0 (IBM Corp., Armonk, NY, USA).

### 2.7. Ethical Considerations

The study was ethically approved by the Ethics Committee of the “Victor Babeș” Clinical Hospital for Infectious Diseases and Pneumophthisiology, Timișoara, Romania (approval no. 11901/16.12.2025). The dataset consisted of anonymized clinical information collected during routine care. No additional consent was required by the Ethics Committee for this retrospective analysis. Written consent for hospitalization and for research use of anonymized data was obtained at admission as part of routine institutional practice. The study adhered to institutional standards and Helsinki principles, in accordance with national requirements.

## 3. Results

### 3.1. Study Population and Cardiovascular Risk Stratification

A total of 395 consecutive adult patients hospitalized with confirmed COVID-19 were included in the final analysis. All patients met diagnostic criteria for SARS-CoV-2 infection confirmed by molecular testing (RT-PCR) or validated antigen testing, were aged ≥18 years, and required hospitalization for acute COVID-19-related illness.

Based on cardiovascular comorbidities present at admission, patients were stratified into three mutually exclusive groups: (1) patients without arterial hypertension or heart failure, (2) patients with arterial hypertension without heart failure, and (3) patients with concomitant arterial hypertension and heart failure. One patient with a history of heart failure in the absence of arterial hypertension was identified but excluded from comparative statistical analyses due to insufficient sample size and was described descriptively only.

The distribution of patients across cardiovascular risk groups was as follows: no arterial hypertension and no heart failure (n = 23, 5.8%), arterial hypertension without heart failure (n = 193, 48.9%), and concomitant arterial hypertension and heart failure (n = 178, 45.1%).

Baseline demographic and clinical characteristics according to cardiovascular risk group are summarized in Table 1.

### 3.2. Baseline Demographic and Clinical Characteristics

Baseline characteristics differed across the three cardiovascular risk groups, as shown in Table 1. Age differed markedly between groups (Kruskal–Wallis test: H = 36.76, *p* < 0.001). Patients with arterial hypertension, particularly those with concomitant heart failure, were substantially older than patients without cardiovascular comorbidities. Median age increased progressively from 59.0 (44.0–68.0) years in patients without arterial hypertension or heart failure to 72.0 (66.0–78.0) years in patients with arterial hypertension alone and 74.0 (68.0–81.0) years in those with both arterial hypertension and heart failure.

The proportion of male patients was similar across groups (60.9% in No HTN/No HF, 50.8% in HTN only, and 51.7% in HTN + HF; *p* = 0.612). BMI was higher in both hypertensive groups (28.4 [25.2–32.1] kg/m^2^ in HTN only and 28.1 [24.8–31.6] kg/m^2^ in HTN + HF) compared with patients without cardiovascular comorbidities (25.8 [22.1–29.4] kg/m^2^).

Vaccination coverage prior to hospitalization was substantial across all cardiovascular risk groups, with 52.2% to 64.8% of patients being vaccinated (≥1 dose) prior to hospitalization. Smoking prevalence was highest among patients without cardiovascular disease and progressively lower among those with increasing cumulative cardiovascular burden.

### 3.3. In-Hospital Clinical Outcomes

In-hospital clinical outcomes according to cardiovascular risk group are presented in Table 2. Overall, in-hospital mortality was 7.3% (29 of 395 patients).

**Figure 1:**
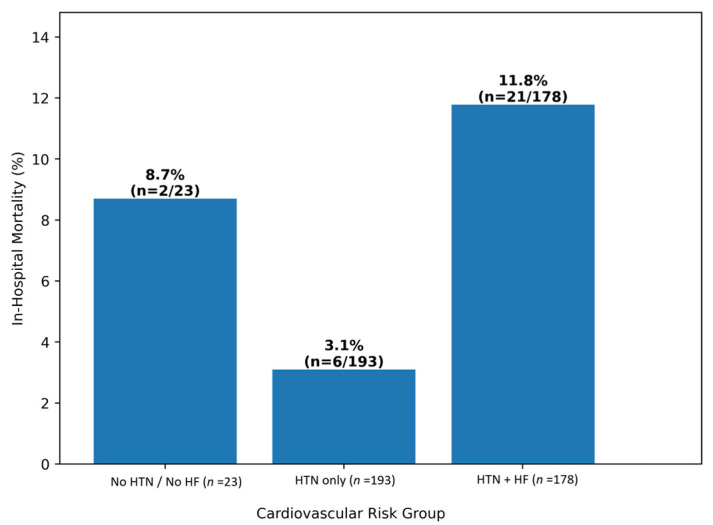
In-hospital mortality across cardiovascular risk groups. Bars represent in-hospital mortality rates (%), with absolute numbers of deaths shown above each bar. A stepwise increase in mortality is observed with increasing cumulative cardiovascular burden, with the highest mortality among patients with concomitant arterial hypertension and heart failure. The higher mortality observed among patients without cardiovascular comorbidities likely reflects the small sample size of this subgroup.

A clear gradient of adverse outcomes was observed across cardiovascular risk strata. The lowest in-hospital mortality rate was recorded among patients with arterial hypertension without heart failure (3.1%), whereas patients without cardiovascular comorbidities had a mortality rate of 8.7%. The highest mortality occurred among patients with concomitant arterial hypertension and heart failure (11.8%). This stepwise increase in mortality with increasing cumulative cardiovascular burden is illustrated in Figure 1.

Length of hospital stay increased progressively across cardiovascular risk groups, from 8.8 ± 5.8 days in patients without arterial hypertension or heart failure to 11.8 ± 7.4 days in patients with arterial hypertension alone and 13.2 ± 9.1 days in those with both arterial hypertension and heart failure (*p* < 0.001). Among patients requiring intensive care, the duration of ICU stay was comparable between patients with arterial hypertension with and without heart failure.

### 3.4. Primary Outcome: In-Hospital Mortality

**Figure 2:**
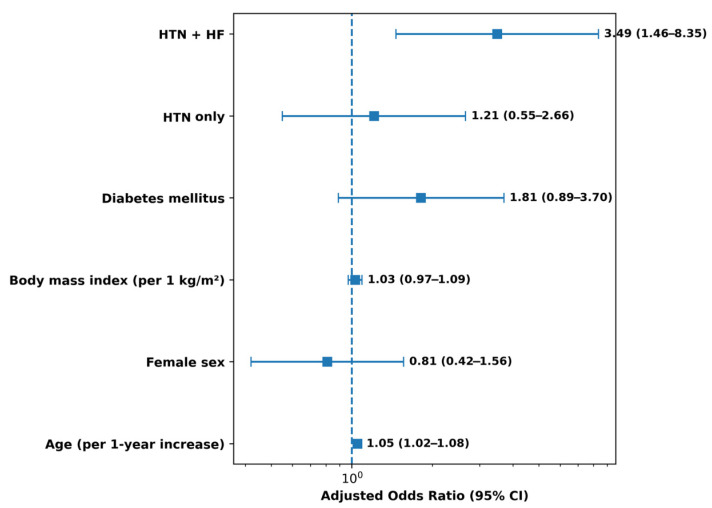
Multivariable adjusted odds ratios for in-hospital mortality. Forest plot showing adjusted odds ratios (ORs) with 95% confidence intervals (CIs) for predictors of in-hospital mortality. The model was adjusted for age, sex, body mass index, diabetes mellitus, and cardiovascular risk group. The vertical dashed line indicates the null effect (OR = 1). For cardiovascular burden categories, the reference group was patients without arterial hypertension and without heart failure (No HTN/No HF).

Group-level comparisons showed higher in-hospital mortality in patients with combined arterial hypertension and pre-existing heart failure, compared with those without this cardiovascular burden. In adjusted analysis, the HTN + HF group had 3.49-fold higher odds of in-hospital death (OR 3.49; 95% CI 1.46–8.35; *p* = 0.004). In contrast, patients with arterial hypertension without heart failure had fewer fatal events, and isolated hypertension did not retain an independent association with in-hospital mortality after adjustment. Adjusted logistic regression results (including age, sex, BMI, diabetes status, and vaccination status as covariates) are reported in Table 3 and summarized in Figure 2.

### 3.5. Secondary Outcomes: ICU Admission and Respiratory Support

ICU admission rates followed a similar gradient to in-hospital mortality across cardiovascular risk groups (Table 2). Among the specified groups, patients with both arterial hypertension and heart failure had the highest ICU admission rate (8.4%), compared with 4.3% in patients without either condition and 2.6% in patients with arterial hypertension alone. Direct comparisons show that those with combined arterial hypertension and heart failure had a significantly increased risk of ICU admission compared with patients with neither condition (odds ratio [OR] 3.24; 95% confidence interval [CI] 1.18–8.88; χ^2^ = 5.15; *p* = 0.023).

Requirements for supplemental oxygen therapy varied by cardiovascular risk group. Oxygen supplementation was more frequently needed in this group, most often in those with arterial hypertension alone, whereas those with both arterial hypertension and heart failure required supplemental oxygen less frequently than those with arterial hypertension alone. Table 2 provides detailed data for each group regarding oxygen therapy and respiratory support.

### 3.6. Laboratory Parameters at Hospital Admission

Inflammatory and thrombotic biomarkers measured at hospital admission are summarized in Table 1. Patients with concomitant arterial hypertension and heart failure exhibited higher levels of interleukin-6 (IL-6) and leukocyte counts compared with patients without cardiovascular comorbidities. Serum creatinine levels were also progressively higher with increasing cumulative cardiovascular burden.

C-reactive protein and D-dimer levels were elevated across all cardiovascular risk groups, reflecting the systemic inflammatory and prothrombotic state characteristic of hospitalized COVID-19 patients. Given the markedly right-skewed distributions of CRP, IL-6, and D-dimer, these biomarkers were summarized using median and interquartile range.

**Figure 3:**
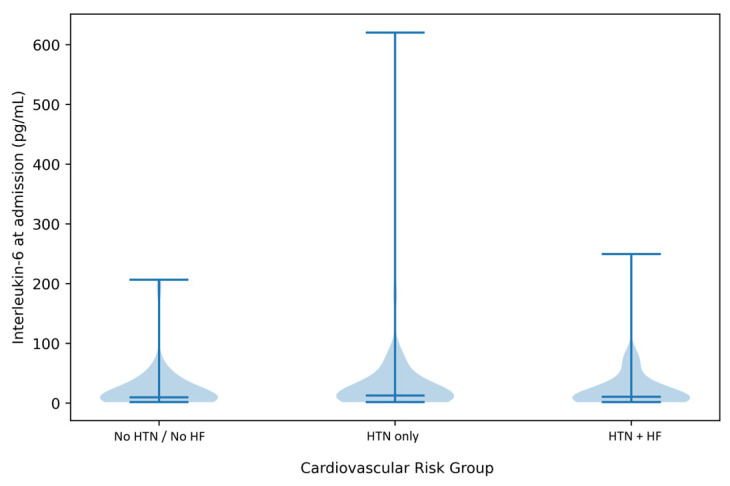
Distribution of interleukin-6 levels at hospital admission according to cardiovascular risk group. Violin plots illustrate the distribution of individual interleukin-6 (IL-6) values measured at admission across cardiovascular risk groups, with median values indicated within each violin. Patients with concomitant arterial hypertension and heart failure show a broader and right-skewed IL-6 distribution compared with patients without arterial hypertension and without heart failure. HTN, arterial hypertension; HF, heart failure.

The distribution of inflammatory and renal biomarkers across cardiovascular risk groups is illustrated in Figure 3, highlighting substantial inter-individual heterogeneity, particularly for IL-6.

### 3.7. Cardiovascular and Infectious Complications

Acute cardiovascular complications documented during hospitalization included arrhythmias, acute decompensated heart failure, and acute myocardial infarction. These complications were more frequently observed in patients with concomitant arterial hypertension and heart failure.

In addition to these cardiovascular complications, systemic anticoagulation was administered to 115 patients (29.1%). Higher-intensity anticoagulation regimens were predominantly used in patients with documented thrombotic complications or prolonged immobilization.

Secondary infectious complications were identified in 38 patients (9.6%), occurring more commonly among those with concomitant arterial hypertension and heart failure. Blood cultures most frequently demonstrated multidrug-resistant Gram-negative organisms, including carbapenem-resistant *Klebsiella pneumoniae* and *Pseudomonas aeruginosa*. Gram-positive pathogens were identified less frequently, including vancomycin-resistant *Enterococcus faecium* and methicillin-resistant Staphylococcus aureus.

## 4. Discussion

In this cohort of 395 adults hospitalized with COVID-19 between March 2020 and December 2024, coexisting arterial hypertension and pre-existing heart failure led to a stepwise rise in short-term in-hospital mortality and ICU admission, compared with either hypertension alone or no cardiovascular disease. This combined cardiovascular burden identified a vulnerable phenotype with higher resource use and more pronounced inflammatory and renal abnormalities at admission. However, our single-centre design and limited number of deaths mean these associations should be interpreted with caution and considered primarily hypothesis-generating.

### 4.1. Cumulative Cardiovascular Burden and COVID-19 Severity

Building on these findings, our results show that patients with both arterial hypertension and heart failure had about 3.5-fold higher adjusted odds of in-hospital mortality (OR 3.49, 95% CI 1.46–8.35). While statistically significant, the wide confidence interval reflects moderate precision and the limited number of outcome events. These findings are thus hypothesis-generating and require validation in larger, multicenter cohorts before firm clinical conclusions can be drawn. This relationship remained significant in models adjusting for age, sex, BMI, diabetes, and vaccination status. The observation supports the idea that cardiovascular multimorbidity increases COVID-19 severity, likely reflecting synergistic vulnerability rather than a simple additive effect [18,19].

This distinction is further supported by the fact that heart failure has consistently led to poor COVID-19 outcomes. Heart failure is linked to impaired cardiocirculatory reserve, higher risk of arrhythmias, and limited ability to tolerate hypoxia or systemic inflammation [18,20]. By contrast, the impact of arterial hypertension alone has varied and is often smaller after adjusting for age and comorbidities. Our findings help explain this by showing that hypertension has major prognostic relevance, mainly when it exists with established cardiac dysfunction. This highlights the need for contextual cardiovascular assessment [21].

### 4.2. Inflammatory Burden and the Role of IL-6

A key mechanistic observation of our study is the link between cumulative cardiovascular disease and increased inflammatory activation. This was evident by elevated, highly variable interleukin-6 (IL-6) levels. Violin plots of individual-level data showed wider, right-skewed distributions of IL-6 in patients with both arterial hypertension and heart failure. This provides biological plausibility for our clinical findings.

This interpretation is supported by prior evidence linking higher IL-6 and CRP levels to respiratory failure and mortality in COVID-19 [22]. Our results also align with earlier work showing that IL-6, cardiac biomarkers, clinical factors, and vaccination status together predict outcomes in COVID-19 sepsis. This highlights the central role of systemic inflammation in disease progression [23].

### 4.3. Pathophysiological Interplay Between Arterial Hypertension, Heart Failure, and SARS-CoV-2 Infection

Turning to pathophysiology, the adverse interaction between arterial hypertension, heart failure, and COVID-19 likely involves overlapping pathways. Both hypertension and heart failure feature endothelial dysfunction, arterial stiffness, neurohormonal activation, and chronic low-grade inflammation. These factors can worsen SARS-CoV-2–induced endothelial injury, microvascular dysfunction, and thromboinflammation [24,25].

Endothelial dysfunction is a key feature of severe COVID-19. It impairs tissue perfusion, causes prothrombotic states, and leads to multiorgan failure [25]. In patients with pre-existing cardiovascular disease, these problems may accelerate cardiac decompensation and worsen systemic injury, especially during cytokine-driven immune dysregulation [26]. This unstable hemostatic environment may cause both thrombotic and hemorrhagic complications, illustrated by reports of deep vein thrombosis and spontaneous soft-tissue hematomas during recovery from SARS-CoV-2 infection [27].

### 4.4. Arterial Hypertension Alone Versus Combined Cardiovascular Disease

Our cohort also revealed a key clinical point: Mortality was lower among patients with only arterial hypertension than in those with combined cardiovascular disease. This supports the idea that arterial hypertension alone is not always high-risk. Its prognostic impact depends on the broader cardiovascular context [21,28].

From a clinical perspective, these results suggest that risk-stratification should go beyond binary classification of comorbidities. Risk assessments should include markers of structural heart disease and overall cardiovascular burden.

### 4.5. ICU Admission, Resource Utilization, and Clinical Trajectory

The stepwise rise in ICU admissions and hospital stays between cardiovascular risk groups highlights the impact of combined hypertension and heart failure. Patients with these combined cardiovascular diseases needed more intensive care and longer hospitalization. This reflects higher disease severity and more complex management [18,19].

ICU duration was similar across groups after admission. This suggests that baseline cardiovascular burden mainly raises the risk of deterioration but may not affect the clinical course once critical illness is established [29].

### 4.6. Strengths and Limitations

This study has several strengths. It uses a real-world design, explicit cardiovascular risk stratification, and integrates laboratory biomarkers with clinical outcomes. Showing individual biomarker levels adds detail beyond summary statistics and helps with mechanistic interpretation.

Several limitations should be acknowledged. First, SARS-CoV-2 variant type and wave classification were not systematically captured and could not be reconstructed for all cases. This prevented us from adjusting for dominant variants (e.g., Delta versus Omicron) or pandemic period effects. Residual confounding from temporal changes in virulence, vaccination coverage, and clinical management cannot be excluded. BNP/NT-proBNP and echocardiographic HF severity parameters were not systematically available; thus, residual confounding related to heart failure severity cannot be excluded. The study is retrospective and single-center. Patients with only heart failure were excluded from comparisons due to small numbers. Detailed echocardiographic phenotyping was not consistently available, preventing distinction between heart failure subtypes [20]. Future studies should include systematic echocardiographic assessment to determine whether HF phenotypes (HFrEF, HFmrEF, HFpEF) confer different levels of risk in COVID-19.

Conducted at a single infectious diseases center in Eastern Europe, our findings primarily reflect the epidemiology, cardiovascular risk profile, and healthcare delivery characteristics of this setting. Extrapolation to other regions and health systems should therefore be made with caution, particularly where baseline cardiovascular risk or COVID-19 management pathways differ. Nonetheless, the high burden of hypertension and heart failure in many Eastern European and low-to middle-income countries suggests that this vulnerable cardiovascular phenotype may be relevant beyond our local context.

### 4.7. Clinical and Research Implications

These findings affect clinical practice. Early identification of patients with both arterial hypertension and heart failure on admission can encourage closer monitoring, timely care escalation, and more personalized management [18,20]. Further validation in independent cohorts is needed, together with studies assessing whether anti-inflammatory or cardioprotective strategies can improve outcomes in this high-risk phenotype [23,30]. Longitudinal data further indicate that cardiometabolic factors (e.g., obesity and metabolic syndrome) may promote progression of left ventricular diastolic dysfunction after COVID-19, supporting the idea of a vulnerable cardiovascular substrate beyond the acute phase [31].

### 4.8. Relevance Across Viral Variants and the Evolving Pandemic Landscape

Although our cohort spans multiple pandemic waves and likely includes patients infected with different SARS-CoV-2 variants (including the ancestral strain, Alpha, Delta, and Omicron sublineages), variant-specific data were not available for analysis. Emerging evidence suggests that Omicron and its sublineages are associated with attenuated pulmonary virulence compared with earlier variants, yet systemic inflammatory responses and cardiovascular complications remain clinically relevant, particularly in patients with pre-existing cardiovascular disease [32,33]. The pathophysiological interplay between chronic cardiovascular conditions (endothelial dysfunction, neurohormonal activation, low-grade inflammation) and acute viral infection is unlikely to be variant-specific; rather, it reflects a fundamental vulnerability of the cardiovascular system to systemic inflammatory stress.

Interleukin-6, a key mediator of the cytokine response, has been implicated in COVID-19 severity across variants and represents a potential therapeutic target in high-risk cardiovascular phenotypes [34,35]. Future studies incorporating variant-specific stratification and serial assessment of inflammatory biomarkers will be essential to determine whether the excess risk conferred by cumulative cardiovascular burden persists—or is attenuated—in the era of Omicron dominance and widespread population immunity [32,33]. These inflammatory and endothelial pathways appear to be conserved across SARS-CoV-2 variants, including Omicron lineages, even though pulmonary virulence has declined in the later pandemic phases [32]. Consequently, patients with pre-existing cardiovascular multimorbidity may remain susceptible to severe systemic inflammation and decompensation despite improved population immunity and changes in viral tropism.

Our findings therefore suggest that cumulative cardiovascular vulnerability represents a variant-agnostic risk amplifier, which persists in the Omicron and vaccinated era and may remain relevant for future COVID-19 surges or similar respiratory viral threats [32,33,34,35].

## 5. Conclusions

In this cohort of hospitalized COVID-19 patients, the combined presence of arterial hypertension and pre-existing heart failure identified patients at increased risk for in-hospital mortality, ICU admission, and prolonged hospitalization. Clinicians should closely monitor patients with both conditions, as these risks were higher compared to those without cardiovascular comorbidities or with only arterial hypertension.

Importantly, arterial hypertension alone was not independently linked to an increase in in-hospital mortality. Its relevance as a prognostic factor in COVID-19 is highly context-dependent and substantially amplified by established cardiac dysfunction. These findings highlight the value of assessing cardiovascular comorbidities holistically, rather than in isolation.

A combined cardiovascular burden was associated with increased inflammatory activation, as evidenced by elevated, variable interleukin-6 levels at hospital admission. These findings highlight the clinical significance of systemic inflammation as a driver of disease severity in patients with preexisting cardiovascular conditions.

Replication in independent cohorts from diverse healthcare systems is warranted. In addition, interventional research should explore whether targeted cardioprotective strategies (for example, optimized guideline-directed heart failure therapy) and anti-inflammatory approaches (including IL-6–directed or broader immunomodulatory regimens where appropriate) can mitigate the excess risk observed in patients with concomitant arterial hypertension and heart failure.

In summary, among hospitalized COVID-19 patients, the coexistence of arterial hypertension and pre-existing heart failure flags a potentially high-risk cardiovascular phenotype within this single-centre cohort, which appears to be associated with higher short-term in-hospital mortality and greater resource utilization than either no cardiovascular disease or hypertension alone. These observations support a cumulative assessment of cardiovascular comorbidities for risk stratification but should be viewed as hypothesis-generating given the single-centre design and the modest number of events. Confirmation in larger, multicentre cohorts is needed before these findings can be translated into definitive prognostic tools or management algorithms.

## Figures and Tables

**Table 1:** Baseline demographic, clinical, comorbidity, chronic medication, and admission laboratory characteristics according to cardiovascular risk group. Data are presented as median (interquartile range), mean ± standard deviation, or n (%), as appropriate.

Variable	No HTN/HF (n = 23)	HTN Only (n = 193)	HTN + HF (n = 178)	*p-*Value
Demographics and clinical presentation				
Age, years	59.0 (44.0–68.0)	72.0 (66.0–78.0)	74.0 (68.0–81.0)	<0.001
Male sex, n (%)	14 (60.9)	98 (50.8)	92 (51.7)	0.612
Urban residence, n (%)	16 (69.6)	124 (64.2)	103 (57.9)	0.214
Body mass index, kg/m^2^	25.8 (22.1–29.4)	28.4 (25.2–32.1)	28.1 (24.8–31.6)	0.041
Current/former smoking, n (%)	9 (39.1)	58 (30.1)	41 (23.0)	0.112
Vaccinated (≥1 dose), n (%)	12 (52.2)	125 (64.8)	98 (55.1)	0.068
Symptom onset before admission, days	5.0 (3.0–7.0)	5.0 (3.0–7.0)	5.0 (3.0–8.0)	0.724
Systolic blood pressure at admission, mmHg	128 (118–138)	142 (130–155)	145 (132–160)	<0.001
Diastolic blood pressure at admission, mmHg	78 (70–85)	82 (75–90)	80 (70–90)	0.092
SpO_2_ at admission, %	93 (89–96)	93 (89–96)	91 (87–95)	0.024
Comorbidities				
Diabetes mellitus, n (%)	5 (21.7)	68 (35.2)	74 (41.6)	0.087
COPD, n (%)	2 (8.7)	24 (12.4)	26 (14.6)	0.641
Chronic kidney disease (CKD), n (%)	1 (4.3)	14 (7.3)	22 (12.4)	0.124
Atrial fibrillation, n (%)	0 (0.0)	18 (9.3)	41 (23.0)	<0.001
Ischemic heart disease, n (%)	1 (4.3)	38 (19.7)	62 (34.8)	<0.001
Admission biomarkers				
CRP at admission, mg/L	98.9 (43.4–141.3)	109.8 (48.8–140.8)	103.1 (49.7–139.7)	0.116
IL-6 at admission, pg/mL	9.5 (3.2–28.1)	12.6 (5.2–33.8)	10.4 (3.8–25.1)	0.053
Creatinine at admission, mg/dL	0.88 (0.74–1.06)	0.94 (0.78–1.22)	1.12 (0.88–1.48)	<0.001
D-dimer, mg/L	0.8 (0.6–1.0)	0.7 (0.5–1.1)	0.8 (0.6–1.2)	0.601
Urea at admission, mg/dL	34 (26–46)	40 (30–58)	52 (36–78)	<0.001
Chronic cardiovascular medication				
Number of antihypertensive drugs	0 (0–0)	2 (1–3)	3 (2–4)	<0.001
ACEi/ARB use, n (%)	2 (8.7)	78 (40.4)	68 (38.2)	<0.001
Beta-blocker use, n (%)	3 (13.0)	68 (35.2)	112 (62.9)	<0.001
In-hospital course (short-term outcomes)				
Length of stay, days	8.8 ± 5.8	11.8 ± 7.4	13.2 ± 9.1	<0.001
ICU admission, n (%)	1 (4.3)	5 (2.6)	15 (8.4)	0.023
In-hospital mortality, n (%)	2 (8.7)	6 (3.1)	21 (11.8)	0.004

Note: Continuous variables are presented as median (IQR), except length of stay and ICU length of stay (mean ± SD), and compared using the Kruskal–Wallis or ANOVA test, as appropriate. Categorical variables are presented as n (%) and compared using the Chi-square or Fisher’s exact test. ACEi/ARB = angiotensin-converting enzyme inhibitors/angiotensin receptor blockers; HTN = arterial hypertension; HF = heart failure.

**Table 2:** Clinical Outcomes and Hospitalization Course by Cardiovascular Risk Group. Data presented as n (%) or mean ± SD. ICU, intensive care unit.

Clinical Outcome	No HTN/HF (n = 23)	HTN Only (n = 193)	HTN + HF (n = 178)	*p-*Value
In-hospital mortality, n (%)	2 (8.7)	6 (3.1)	21 (11.8)	0.004
ICU admission, n (%)	1 (4.3)	5 (2.6)	15 (8.4)	0.023
Supplemental oxygen therapy, n (%)	0 (0.0)	31 (16.1)	18 (10.1)	0.018
Length of hospital stay, days	8.8 ± 5.8	11.8 ± 7.4	13.2 ± 9.1	<0.001
ICU length of stay, days	—	8.0 ± 6.5	8.9 ± 7.0	0.71

**Table 3:** Multivariable Logistic Regression Analysis for In-Hospital Mortality.

Variable	Adjusted OR	95% Confidence Interval	*p* Value
Age (per 1-year increase)	1.05	1.02–1.08	0.001
Female sex	0.81	0.42–1.56	0.53
Body mass index (per 1 kg/m^2^)	1.03	0.97–1.09	0.31
Diabetes mellitus	1.81	0.89–3.70	0.10
HTN only	1.21	0.55–2.66	0.63
HTN + HF	3.49	1.46–8.35	0.004

## Data Availability

Deidentified clinical, laboratory, and imaging data supporting the findings of this study are available from the corresponding authors upon reasonable request, subject to institutional data-sharing policies.

## Abbreviations

The following abbreviations are used in this manuscript:

COVID-19	Coronavirus disease 2019
SARS-CoV-2	Severe acute respiratory syndrome coronavirus 2
HTN	Arterial hypertension
HF	Heart failure
ICU	Intensive care unit
BMI	Body mass index
CRP	C-reactive protein
IL-6	Interleukin-6
RT-PCR	Reverse transcription polymerase chain reaction
NYHA	New York Heart Association
OR	Odds ratio
CI	Confidence interval
SD	Standard deviation
IQR	Interquartile range
